# Aseptic Failure Rates in Revision Total Knee Arthroplasty Using Structural Allografts Versus Porous Metal Cones for Anderson Orthopaedic Research Institute (AORI) Grade 2 and 3 Defects: A Systematic Review and Meta-Analysis of Non-randomized Studies

**DOI:** 10.7759/cureus.113772

**Published:** 2026-08-01

**Authors:** Jorge Rojas, German Rojas, Maria Quintero Rojas, Gamal Zayed, Klaus Mieth, Diego Alarcon Perico

**Affiliations:** 1 Department of Orthopedics and Traumatology, Hospital Universitario Fundación Santa Fe de Bogotá, Bogotá, COL; 2 Department of Orthopedics and Traumatology, Hospital Serena del Mar, Cartagena, COL

**Keywords:** aori grade 2, aori grade 3, aseptic loosening, bone loss, porous metal cones, re-revision rates, revision total knee arthroplasty, structural allografts

## Abstract

Management of significant bone loss in revision total knee arthroplasty (rTKA) poses unique challenges. This systematic review and meta-analysis evaluates the effectiveness of structural allografts and porous metal cones for Anderson Orthopaedic Research Institute (AORI) Grade 2 and 3 defects, focusing on their re-revision rates due to aseptic loosening. Following Preferred Reporting Items for Systematic Reviews and Meta-Analyses (PRISMA) guidelines, databases including MEDLINE, EMBASE, and CINAHL were searched up to June 2023. We included original clinical studies (January 2000-June 2023) assessing structural allografts or porous metal cones in rTKA for AORI 2 or 3 defects. Data from 52 studies (2,568 cases) were analyzed using a random-effects model to calculate pooled re-revision rates due to aseptic loosening. Subgroup analyses were conducted among the metal cones studies to explore heterogeneity sources. The pooled incidence of re-revision due to aseptic loosening was significantly lower for porous metal cones at 0.71% (95% CI: 0.33%-1.51%) compared to 2.81% (95% CI: 1.11%-6.92%) for allografts. Subgroup analysis indicated that re-revision rates for metal cones remained consistently low across varying follow-up durations, affirming their sustained effectiveness. Variability in re-revision rates by study size suggested higher incidences in larger studies, possibly reflecting the complexity of cases or differences in clinical practices. Temporal trend analysis highlighted an increasing preference for metal cones since 2012. Porous metal cones demonstrate superior mid-term durability with significantly lower re-revision rates compared to structural allografts for severe metaphyseal bone defects. Long-term functional outcomes and patient-specific recommendations are necessary to further validate these findings.

## Introduction and background

Revision total knee arthroplasty (rTKA) holds unique challenges, notably the management of significant bone loss classified as grade 2 and 3 defects in the Anderson Orthopaedic Research Institute (AORI) classification [1]. The appropriate management of these defects is crucial for the long-term success of the rTKA [2].

Historically, structural allografts and porous metal cones have been pivotal in managing these defects. However, debate persists regarding which method ensures more durable fixation and minimizes re-revisions from aseptic failure [3]. Considering the significant clinical implications and associated costs of re-revisions, it is crucial to determine whether structural allografts or porous metal cones provide more lasting and effective outcomes [4].

Previous reviews have offered insights into this debate [5,6]; however, with the rapidly evolving evidence, an updated systematic review and meta-analysis is warranted. This study aims to review and analyze current literature, specifically focusing on aseptic re-revision rates as the primary outcome. Furthermore, a temporal trend analysis will be undertaken to understand the shift in surgeons' preference over the past decade concerning these two treatment strategies. By providing a robust comparative analysis of structural allografts versus porous metal cones, this study seeks to guide surgeons in making evidence-based decisions in their clinical practice and ultimately improve patient outcomes in rTKA for AORI grade 2 and 3 defects.

## Review

Methods

This review adheres to the Cochrane Handbook for Systematic Reviews of Interventions and follows the Preferred Reporting Items for Systematic Reviews and Meta-Analyses (PRISMA) framework [7]. Approval was granted by the Ethics Committee (CCEI-16529-2024). 

Eligibility Criteria

Articles were eligible if they: (1) were original clinical studies published in English since January 2000; (2) assessed outcomes of structural allografts or porous metal cones for rTKA addressing AORI grade 2 or 3 bone defects, with a follow-up of at least 12 months; and (3) provided data on re-revision rates due to aseptic loosening or explicitly stated the absence of such re-revisions. We included studies describing bone loss severity equivalent to AORI grade 2 or 3 prior to its widespread adoption. Studies had to report outcomes separately for each intervention; those mixing primary and revision TKA outcomes, or amalgamating different treatment methodologies without clear subgroup outcomes, were excluded. Studies focused solely on oncologic resections or complex primary TKAs were also excluded.

Search Strategy and Study Selection Process

We searched MEDLINE, EMBASE, and CINAHL databases up to June 30, 2023. Search terms included “revision TKA,” “structural allografts,” and “porous metal cones.” Details of the search strategy are available in Appendices 1, 2. Two reviewers independently screened titles and abstracts, then full texts, resolving disagreements through a third reviewer.

Assessment of Study Quality and Risk of Bias

Applicability and methodological quality were assessed using a structured checklist developed for this review, built on the two-domain framework (applicability and methodological quality) described by Luijken et al. for comparative studies of operative interventions, which itself draws on elements of the Cochrane Risk of Bias 2 (RoB 2) tool, ROBINS-I, and the Methodological Index for Non-Randomized Studies (MINORS) [8]. Studies were assessed for applicability and methodological quality across predefined domains, details of which are outlined in Appendices 1, 2.

Data Extraction

Data abstraction was conducted by three independent reviewers using a standardized form to capture study details, patient demographics, intervention specifics, and follow-up periods. Disagreements were resolved through discussion, with a fourth reviewer available to arbitrate unresolved issues.

Data Analysis and Synthesis

Primary outcome measure: The primary outcome was the rate of re-revision surgery due to aseptic loosening, defined as any procedure requiring replacement or removal of the allograft and its associated components or the porous metal cone, excluding modular exchanges and minor procedures that do not significantly alter the components. Re-revisions due to periprosthetic joint infection (PJI) were also excluded. This metric aims to evaluate the durability and longevity of structural allografts versus porous metal cones.

Statistical Analysis

The pooled failure rate for allograft and porous metal cone interventions was calculated using a proportion meta-analysis in Stata software (version 14; StataCorp LLC, College Station, TX, USA). A binomial distribution was used to model within-study variability, particularly because several studies reported a 0% re-revision rate due to aseptic loosening. Confidence intervals (CIs) for each study were determined using the exact method. A random-effects model was applied to accommodate anticipated clinical and methodological heterogeneity. Heterogeneity among studies was quantified using the I^2^ statistic, with values of 0%, 25%, 50%, and 75% indicating no, low, moderate, and high heterogeneity, respectively. 

Temporal Trend Analysis

A longitudinal analysis was conducted to examine the usage trends of allografts and metal cones from January 2000 to June 2023. This analysis charted the annual number of studies reporting on each treatment strategy, reflecting shifts in clinical practice.

Funding

No funds, grants, or other support were received during the preparation of this manuscript. There was no external source of funding for this article.

Results

Search Results

**Figure 1:**
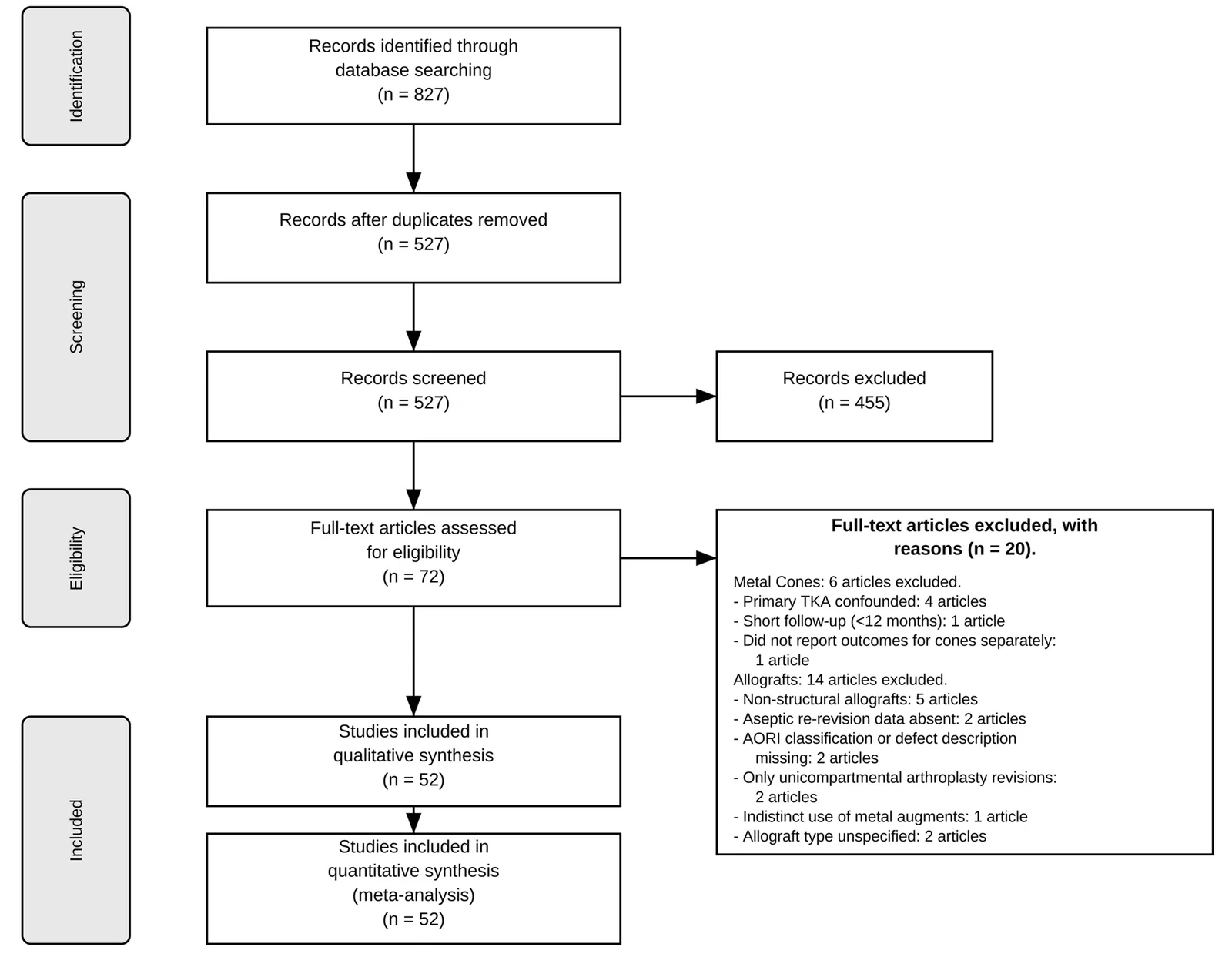
Study selection flow diagram. This flow diagram illustrates the process of selecting studies for inclusion in the review.

The literature search identified 827 potentially relevant titles, of which 527 underwent abstract screening. This led to 72 articles being reviewed in full, resulting in 52 studies included for analysis, as illustrated in Figure 1.

Included Studies

The 52 studies involved a total of 2568 rTKA cases using porous metal cones or structural allografts. No direct comparisons between the two treatments were provided; each study reported outcomes for only one type. The publication span was from 2001 to 2023, primarily consisting of case series and retrospective cohorts with evidence levels III to IV. Data collection was retrospective in 96% of studies and prospective in 4%, covering multiple geographic regions including North America, South America, Europe, and Asia. Median sample size was 38 cases, ranging from 8 to 163. All studies were hospital-based, using either convenience or consecutive sampling. Detailed characteristics are presented in Table 1.

**Table 1:** Characteristics of the included studies.

Study	Year	Country	Data-collection	Number of patients	Mean age of patients (years)	Number of cones/allografts	Type of cone	Mean follow-up (months)
Metal cones								
Abdelaziz et al. [9]	2020	Germany	Ambispective	72	70	101	Zimmer trabecular metal cones	49.9
Abdelaziz et al. [10]	2019	Germany	Retrospective	25	65	32	Zimmer trabecular metal cones	126.5
Baek et al. [11]	2022	USA	Retrospective	49	69	56	Zimmer trabecular metal cones	40.5
Batinica et al. [12]	2022	Italy	Retrospective	60	67.9	94	Zimmer trabecular metal cones	43.5
Bohl et al. [13]	2018	USA	Retrospective	163	67	163	Stryker 2nd generation 3D-printed	30
Burastero et al. [14]	2018	USA - Italy	Retrospective	18	73	26	Zimmer trabecular metal cones	72
Chalmers et al. [15]	2021	USA	Prospective	62	66	77	Stryker 2nd generation 3D-printed	27
Cherny et al. [16]	2021	Canada	Retrospective	29	70	33	Zimmer trabecular metal cones	33
De Martino et al. [17]	2015	France - USA	Retrospective	61	60.35	66	Zimmer trabecular metal cones	24
De Martino et al. [18]	2023	France	Retrospective	51	68	71	Zimmer trabecular metal cones	34
Denehy et al. [19]	2019	USA	Retrospective	24	64	24	Zimmer trabecular metal cones	33
Derome et al. [20]	2014	France	Retrospective	66	73.4	93	Zimmer trabecular metal cones	112
Erivan et al. [21]	2021	Denmark	Retrospective	36	69	38	Zimmer trabecular metal cones	47
Fosco et al. [22]	2013	USA	Retrospective	63	67	66	Zimmer trabecular metal cones	70
Gibon et al. [23]	2022	USA	Retrospective	15	66.1	18	Zimmer trabecular metal cones	31
Girerd et al. [24]	2016	Brazil	Ambispective	10	71.1	12	Zimmer trabecular metal cones	34.7
Howard et al. [25]	2011	Germany	Retrospective	52	68.6	52	Zimmer trabecular metal cones	22
Jacquet et al. [26]	2021	USA	Retrospective	157	64	159	Zimmer trabecular metal cones	60
Jensen et al. [27]	2014	India	Retrospective	48	66	68	Zimmer trabecular metal cones	107
Kamath et al. [28]	2015	United Kingdom	Retrospective	26	72	29	Zimmer trabecular metal cones	36
Kotrych et al. [29]	2022	United Kingdom	Retrospective	14	71	14	Zimmer trabecular metal cones	87
Lachiewicz et al. [30]	2012	Germany	Retrospective	38	72	38	Zimmer trabecular metal cones	37
Long et al. [31]	2009	Spain	Retrospective	21	73	29	Zimmer trabecular metal cones	36
Meneghini et al. [32]	2008	USA	Retrospective	139	66	202	Stryker 2nd generation 3D-printed	28.8
Monarréz et al. [33]	2022	Korea	Retrospective	13	73.2	15	Zimmer trabecular metal cones	82.8
Mozella et al. [34]	2014	USA	Retrospective	107	64.7	107	Zimmer trabecular metal cones	29
Nikolaus et al. [35]	2017	Italy	Retrospective	24	72	31	Zimmer trabecular metal cones	132
Ohlmeier et al. [36]	2021	New Zealand	Retrospective	44	70,4	51	Zimmer trabecular metal cones	48
Panda et al. [37]	2018	Italy	Retrospective	101	68.6	139	Zimmer trabecular metal cones	90
Potter et al. [38]	2016	India	Retrospective	11	63.8	22	Zimmer trabecular metal cones	57
Rajgopal et al. [39]	2019	USA	Retrospective	157	No information	159	Zimmer trabecular metal cones	60
Rajgopal et al. [40]	2021	USA	Retrospective	27	64.6	33	Zimmer trabecular metal cones	39.6
Rao et al. [41]	2013	USA	Retrospective	15	68.1	15	Zimmer trabecular metal cones	34
Remily et al. [42]	2021	Poland	Retrospective	64	71	64	Stryker 2nd generation 3D-printed	28
Rossi et al. [43]	2022	Italy	Retrospective	10	62	12	Zimmer trabecular metal cones	39.8
Schmitz et al. [44]	2013	India	Retrospective	59	69,7	80	Zimmer trabecular metal cones	83
Sandiford et al. [45]	2017	Russia	Retrospective	31	63	35	Stryker 2nd generation 3D-printed	24
Shichman et al. [46]	2022	Argentina	Retrospective	35	66.1	49	Zimmer trabecular metal cones	32.1
Tetreault et al. [47]	2020	Netherlands	Retrospective	123	66.1	156	Zimmer trabecular metal cones	36
Villanueva-Martínez et al. [48]	2013	USA	Retrospective	54	65	68	Stryker 2nd generation 3D-printed	29.9
Garcia-Mansilla et al. [49]	2021	USA	Retrospective	62	64	62	Stryker 2nd generation 3D-printed	40
Allografts								
Sandiford et al. [45]	2017	Canada	Retrospective	30	66	30	Not applicable	109
Backstein et al. [50]	2006	Canada	Retrospective	58	73.4	68	Not applicable	64.8
Bauman et al. [51]	2009	USA	Retrospective	65	67.9	87	Not applicable	90
Burnett et al. [52]	2009	USA	Retrospective	8	67.3	10	Not applicable	48
Chuang et al. [53]	2022	Korea	Retrospective	27	67.96	38	Not applicable	107
Chun et al. [54]	2014	Brasil	Retrospective	25	70	34	Not applicable	55
Clatworthy et al. [55]	2001	USA	Retrospective	46	67	46	Not applicable	97
Cobra et al. [56]	2013	USA	Retrospective	15	59	15	Not applicable	64.8
Engh et al. [57]	2007	Taiwan	Retrospective	28	69.7	50	Not applicable	76
Lyall et al. [58]	2009	Taiwan	Retrospective	12	68.1	12	Not applicable	62.5
Wang et al. [59]	2013	Canada	Retrospective	50	68	66	Not applicable	96.9

Porous Metal Cones

Porous metal cones were evaluated in 41 studies involving 2236 patients and 2568 cones [9-49]. The patient demographics predominantly featured a female majority (range 30.5% to 92.3%) and an average age of 68 years (range 24 to 92 years). A significant majority of the cones used, 83% (34 studies, 1959 cones), were Zimmer® trabecular metal cones (Zimmer Biomet, Inc., Warsaw, Indiana, USA). The remaining 17% (7 studies, 609 cones) were Stryker® second-generation 3D-printed titanium cones (Stryker Corporation, Kalamazoo, Michigan, USA), a technology that has been included since 2019. The analysis indicated a predominant use of tibial cones over femoral cones, highlighting a greater necessity for tibial reconstruction within these cohorts. The primary clinical indication for using porous metal cones was aseptic loosening, followed by issues such as periprosthetic joint infection (PJI), instability, and periprosthetic fractures.

Various prosthetic designs were paired with these metal cones, including posterior-stabilized, constrained condylar, and hinged designs, in both pure and rotating forms. The studies reported an average follow-up duration of 53 months, with a range from 22 to 132 months, providing a moderate to long-term perspective on the performance of metal cones in rTKA scenarios.

Structural Allografts

Structural allografts were assessed in 11 studies encompassing 364 patients and 456 grafts [45,50-59]. The demographic profile was similar to that seen in metal cone studies, with a majority of female patients (range 40.4% to 80.8%) and an average age of 66 (range 26 to 92 years). The allografts primarily originated from femoral heads, frequently fresh-frozen and tailored to specific defect sites. Other sources included humeral heads, proximal tibias, and distal femurs.

Most allografts targeted tibial deficiencies, similar to the usage seen with metal cones. The main reasons for employing allografts were akin to those in metal cone applications, including aseptic loosening and periprosthetic joint infection (PJI). The types of prostheses used with allografts included posterior-stabilized, constrained condylar, and hinged designs, addressing various degrees of joint instability and bone loss. Notably, the average follow-up period in allograft studies was 83 months (ranging from 48 to 109 months), which was significantly longer than the 53 months reported in metal cone studies. This extended follow-up suggests a deeper longitudinal evaluation of the efficacy and durability of structural allografts in rTKA scenarios.

Risk of Bias

**Figure 2:**
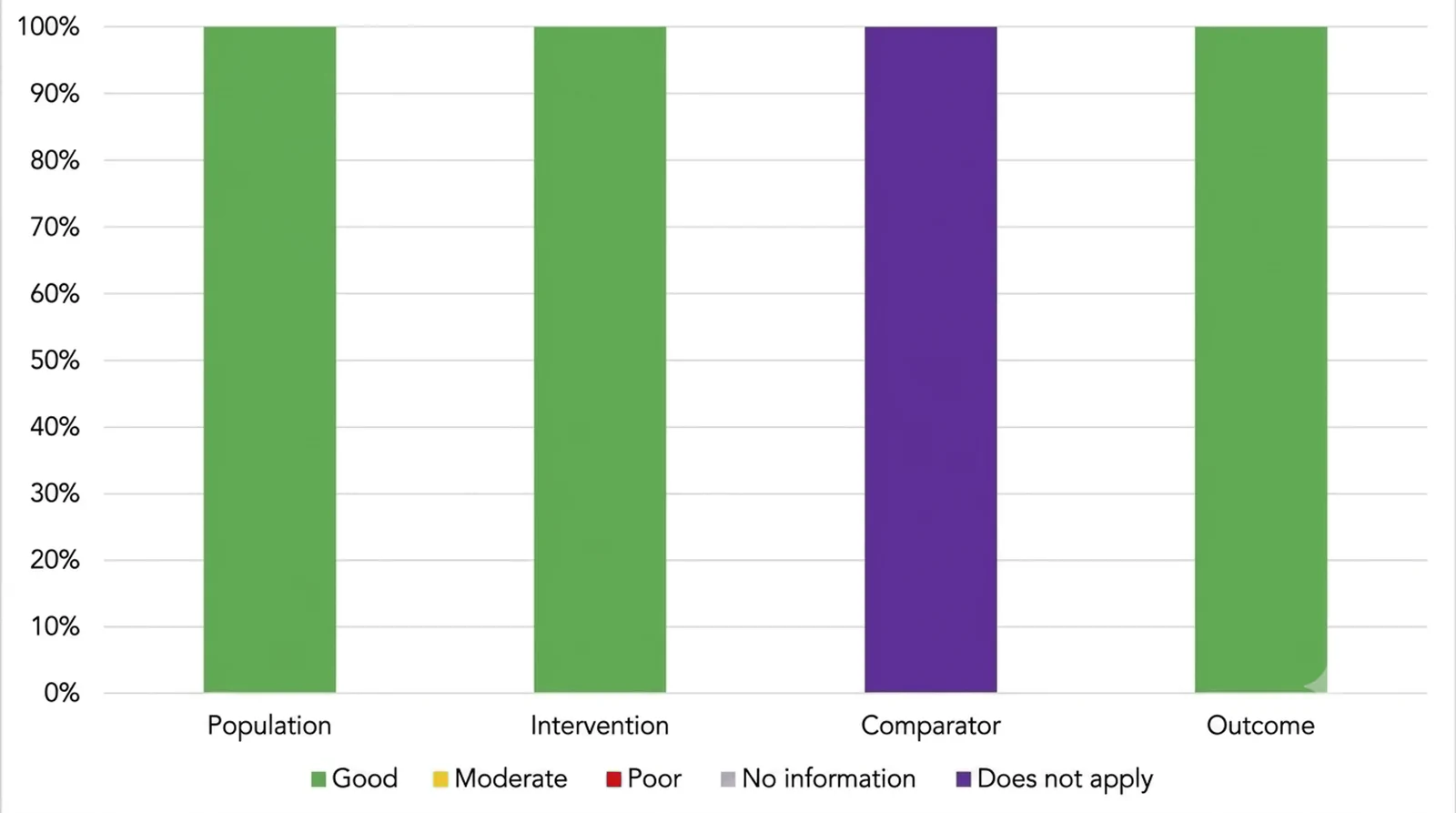
Applicability assessment of included studies. This figure presents the applicability of results from the 52 included studies, evaluated using the Population, Intervention, Comparator, Outcome (PICO) framework. The chart categorizes each domain by the quality of information: Good, Moderate, Poor, No Information, and Does Not Apply. The assessment indicates that all studies had good applicability for Population, Intervention, and Outcome domains. The Comparator domain is marked as "Does Not Apply" for all studies, reflecting the absence of comparative studies in this review.

Part 1-Assessment of applicability of studies: As shown in Figure 2, the majority of studies demonstrated high applicability for population and intervention descriptions, with over 90% rated as 'Good'. Outcome reporting varied, with many rated 'Good' and a significant portion 'Moderate'.

**Figure 3:**
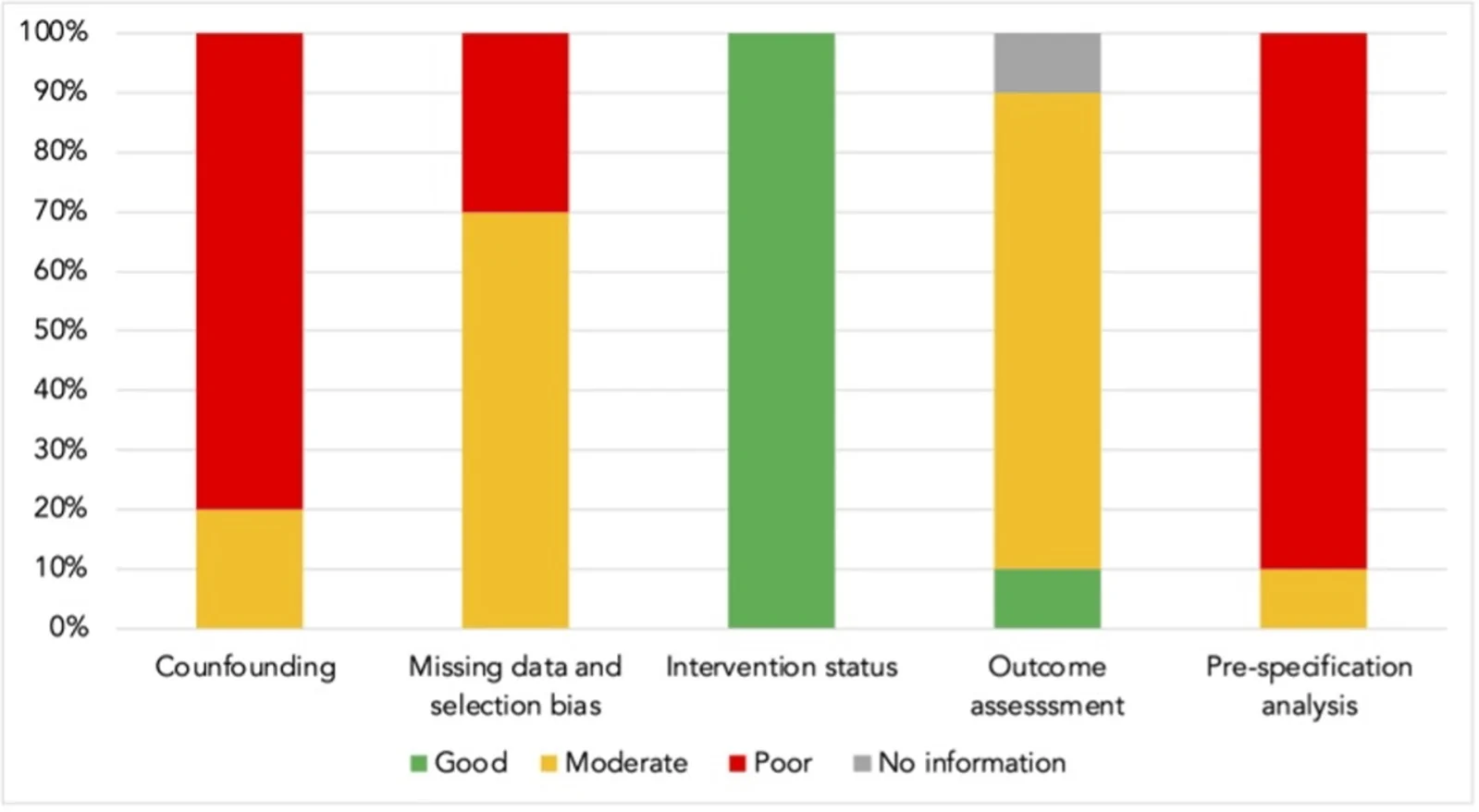
Methodological quality assessment of included studies. This figure presents the methodological quality assessment of the included studies across five domains: Confounding, Missing data and selection bias, Intervention status, Outcome assessment, and Pre-specification analysis. The assessment was conducted using the risk-of-bias tool developed by Luijken et al [8]. The quality of each domain is categorized as Good, Moderate, Poor, or No Information. The results indicate significant variability in the methodological quality, with many studies showing poor handling of confounding and pre-specification analysis, while intervention status is consistently rated as good.

Part 2-Assessment of methodological quality of studies: Figure 3 reveals significant concerns about confounding, with most studies rated 'Poor'. Issues with missing data and selection bias were frequently rated 'Moderate'. While intervention details were generally well reported ('Good'), outcome assessment and analysis plans were uneven, affecting potential outcome reliability.

Primary Outcome: Re-revision Due to Aseptic Loosening

**Figure 4:**
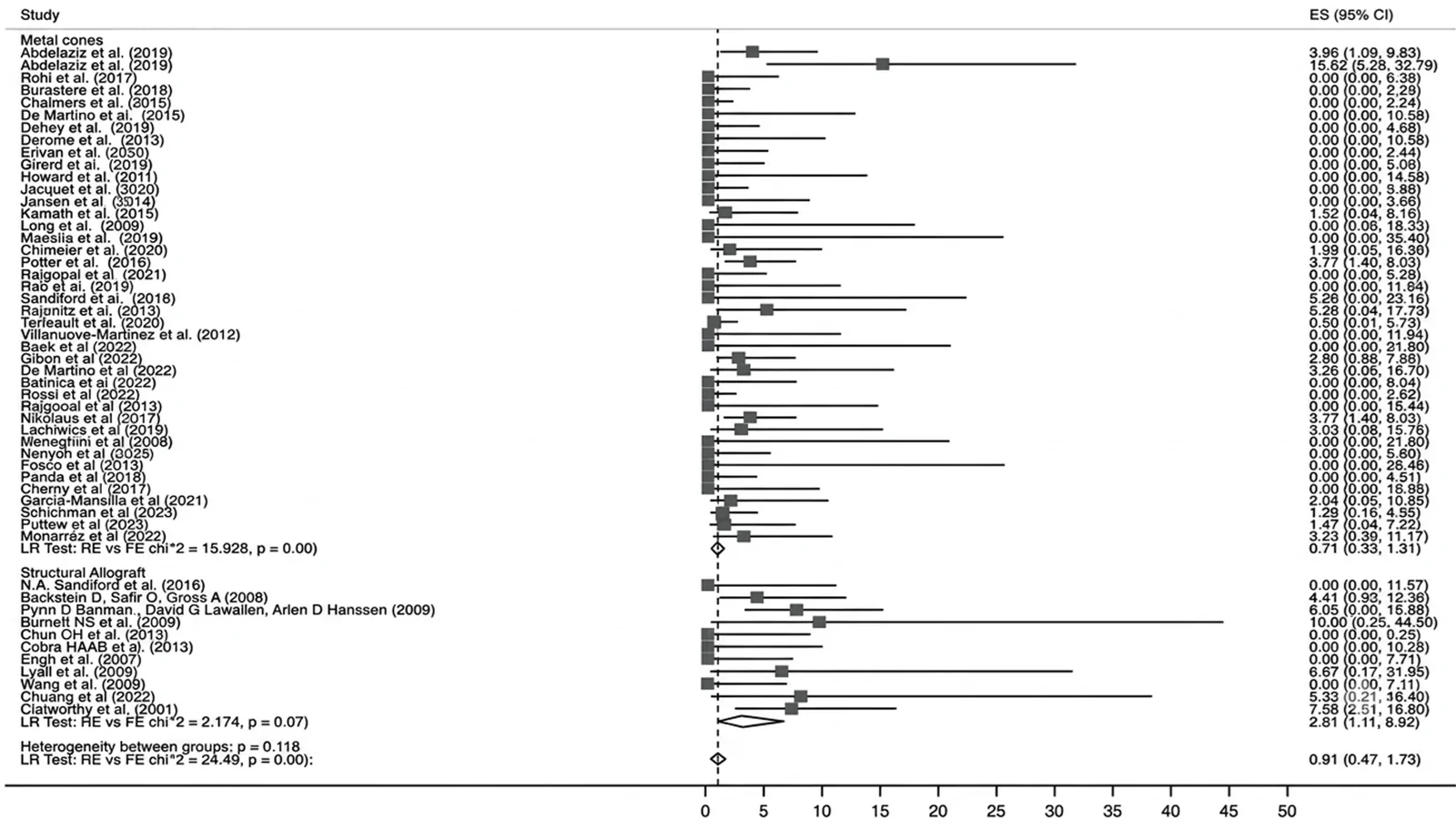
Forest plot showing the individual study effect sizes (ES) and 95% confidence intervals (CIs) for the incidence of re-revision due to aseptic loosening in patients receiving metal cones and structural allografts. Each line represents an individual study with the point estimate of the effect size and the horizontal lines representing the 95% CI. The diamond represents the random pooled effect size across all studies, with its width corresponding to the pooled 95% CI. The ES is measured as the incidence proportion. The left-hand column lists the names of the studies categorized by the type of intervention. Metal cones: Abdelaziz et al. [9], Abdelaziz et al. [10], Baek et al. [11], Batinica et al. [12], Bohl et al. [13], Burastero et al. [14], Chalmers et al. [15], Cherny et al. [16], De Martino et al. [17], De Martino et al. [18], Dehey et al. [19], Derome et al. [20], Erivan et al. [21], Fosco et al. [22], Gibon et al. [23], Girerd et al. [24], Howard et al. [25], Jacquet et al. [26], Jensen et al. [27], Kamath et al. [28], Kotrych et al. [29], Lachiwicz et al. [30], Long et al. [31], Meneghini et al. [32], Monarréz et al. [33], Mozella et al. [34], Nikolaus et al. [35], Ohlmeier et al. [36], Panda et al. [37], Potter et al. [38], Rajgopal et al. [39], Rajgopal et al. [40], Rao et al. [41], Remily et al. [42], Rossi et al. [43], Schmitz et al. [44], Sandiford et al. [45], Shichman et al. [46], Tetreault et al. [47], Villanueva-Martínez et al. [48], García-Mansilla et al. [49]. Structural allografts: Sandiford et al. [45], Backstein et al. [50], Bauman et al. [51], Burnett et al. [52], Chuang et al. [53], Chun et al. [54], Clatworthy et al. [55], Cobra et al. [56], Engh et al. [57], Lyall et al. [58], Wang et al. [59]. The heterogeneity within each subgroup and across the overall analysis is indicated by the Tau-squared statistic, and the p-value for the heterogeneity test is reported. The graph demonstrates a lower pooled incidence of re-revision for metal cones compared to structural allografts.

The meta-analysis included 52 studies evaluating re-revision due to aseptic loosening in patients with porous metal cones and structural allografts (Figure 4). The pooled incidence of re-revision was lower for porous metal cones at 0.71% (95% CI: 0.33% to 1.51%, Tau-squared = 1.16, p < 0.001) compared to 2.81% for structural allografts (95% CI: 1.11% to 6.92%, Tau-squared = 0.65, p = 0.07). There was considerable heterogeneity within the individual treatment groups, as indicated by the Tau-squared statistics, though the heterogeneity within the structural allograft group was not statistically significant. The overall heterogeneity was also pronounced (Tau-squared = 1.37, p < 0.001), indicating significant variability in the reported outcomes.

Subgroup Analysis

Subgroup analyses were performed to identify sources of the observed heterogeneity in studies evaluating porous metal cones. The analyses focused on follow-up duration and study size.

Follow-up Duration

**Figure 5:**
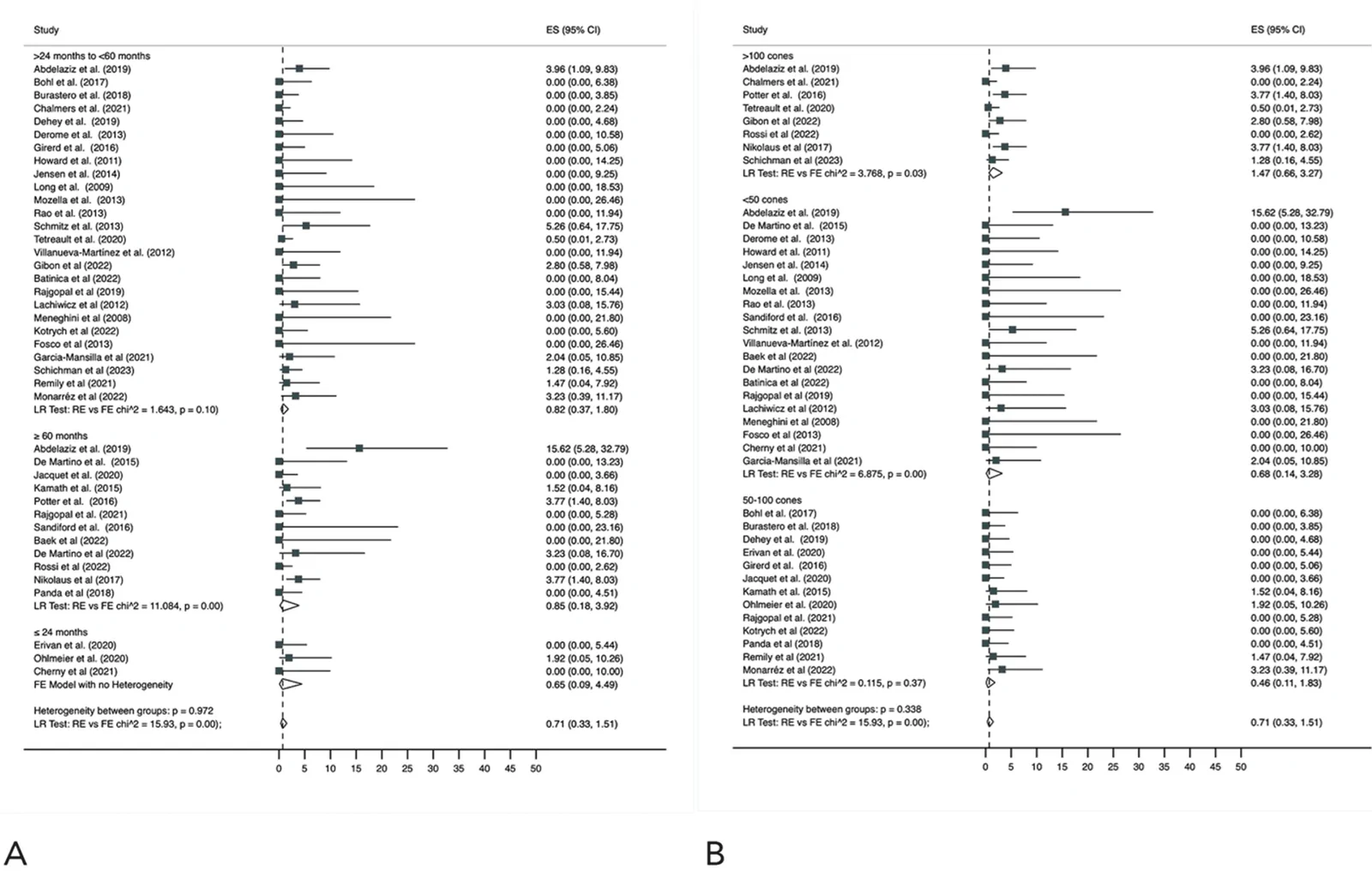
Subgroup analyses of the incidence of re-revision due to aseptic loosening in studies evaluating porous metal cones. (A) subgroup analysis based on the length of follow-up: >24 months to <60 months, ≥60 months, and ≤24 months. (B) Subgroup analysis based on study size: >100 cones, <50 cones, and 50–100 cones. Each study within the subgroups is represented by a point estimate and 95% confidence interval on a forest plot. The pooled effect size (ES) for each subgroup is displayed along with the test for heterogeneity. Studies evaluating porous metal cones: Abdelaziz et al. [9], Abdelaziz et al. [10], Baek et al. [11], Batinica et al. [12], Bohl et al. [13], Burastero et al. [14], Chalmers et al. [15], Cherny et al. [16], De Martino et al. [17], De Martino et al. [18], Dehey et al. [19], Derome et al. [20], Erivan et al. [21], Fosco et al. [22], Gibon et al. [23], Girerd et al. [24], Howard et al. [25], Jacquet et al. [26], Jensen et al. [27], Kamath et al. [28], Kotrych et al. [29], Lachiwicz et al. [30], Long et al. [31], Meneghini et al. [32], Monarréz et al. [33], Mozella et al. [34], Nikolaus et al. [35], Ohlmeier et al. [36], Panda et al. [37], Potter et al. [38], Rajgopal et al. [39], Rajgopal et al. [40], Rao et al. [41], Remily et al. [42], Rossi et al. [43], Schmitz et al. [44], Sandiford et al. [45], Schichman et al. [46], Tetreault et al. [47], Villanueva-Martínez et al. [48], García-Mansilla et al. [49].

Studies were grouped by follow-up periods: less than 24 months, between 24 and 60 months, and more than 60 months. The pooled incidence rates of re-revision due to aseptic loosening were 0.65% (95% CI: 0.09% to 4.49%), 0.82% (95% CI: 0.37% to 1.80%), and 0.85% (95% CI: 0.18% to 3.92%), respectively (Figure 5A). Although heterogeneity was reduced within these subgroups, it was still present, suggesting follow-up duration partly explains the observed overall heterogeneity.

Study Size

Studies were categorized into three groups based on cone counts: fewer than 50 cones, 50 to 100 cones, and more than 100 cones. Incidences of re-revision were 0.68% (95% CI: 0.14% to 3.28%), 0.46% (95% CI: 0.11% to 1.83%), and 1.47% (95% CI: 0.66% to 3.27%), respectively (Figure 5B). Moderate to high heterogeneity persisted, particularly in smaller and larger study groups, indicating that study size also impacts outcome variability. These analyses suggest that while follow-up duration and study size influence heterogeneity, other unmeasured factors may also contribute.

Temporal Trend Analysis

**Figure 6:**
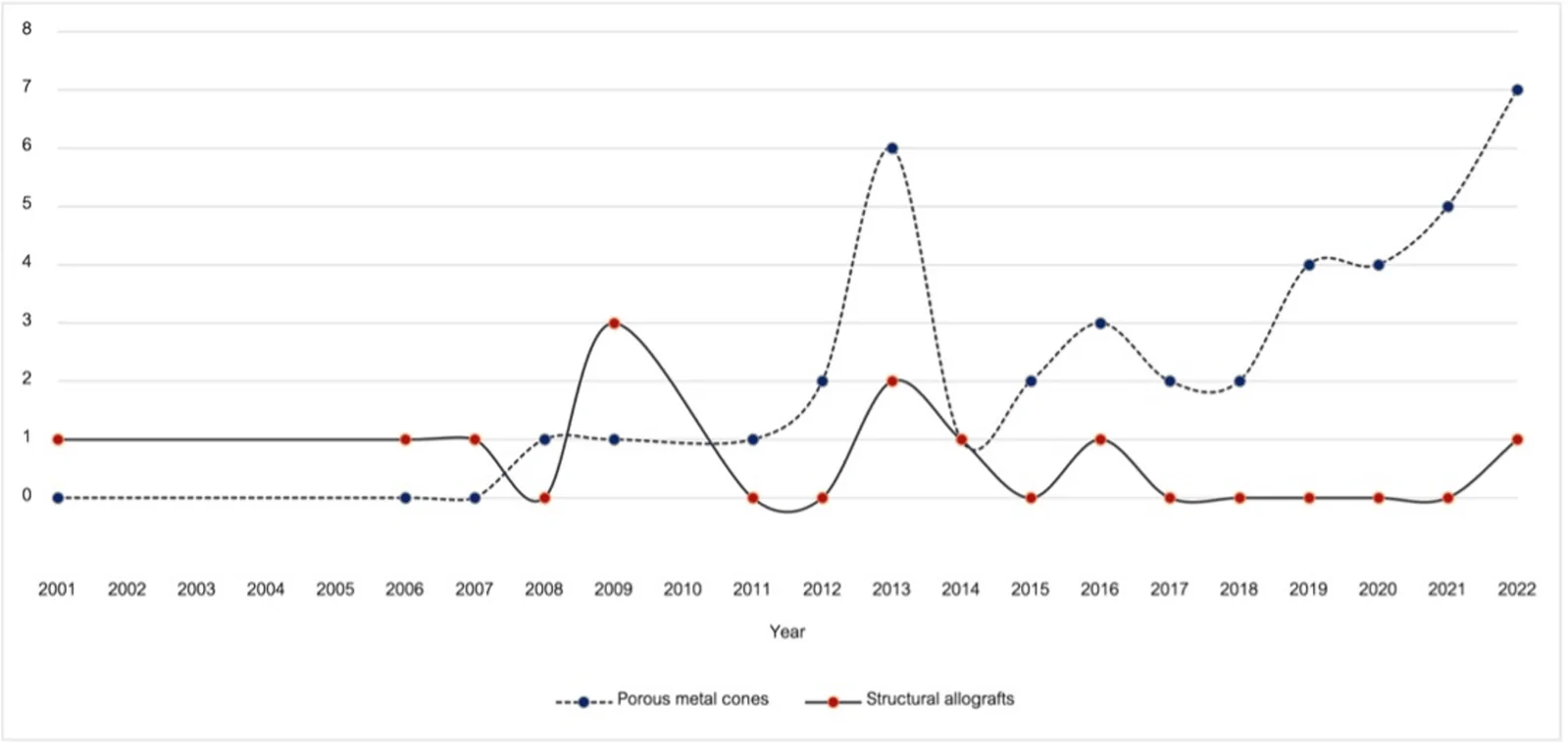
Annual trends in the utilization of porous metal cones and structural allografts for revision total knee arthroplasty (rTKA): a comparative analysis from 2001 to 2022. The dotted line with blue markers represents the number of studies per year involving porous metal cones, showing a general increase in their use over time, particularly from 2012 onwards. The solid line with red markers indicates the number of studies per year focusing on structural allografts, which demonstrates fluctuation over the years with no clear trend. Both lines highlight the evolving landscape of rTKA management options as reported in the literature.

Analysis from 2001 to 2022 showed an increasing preference for porous metal cones, particularly after 2012, while interest in structural allografts peaked in 2009 and then stabilized (Figure 6).

Discussion

The management of significant bone loss during rTKA remains a complex challenge that significantly impacts patient outcomes. This systematic review and meta-analysis consolidates findings from 52 studies, representing a substantial advancement in understanding revision total knee arthroplasty (rTKA) for managing AORI grade 2 and 3 defects. Our analysis, which included over 2568 rTKA cases, reveals a lower incidence of re-revision due to aseptic loosening with porous metal cones (0.71%) compared to structural allografts (2.81%). This extensive collection of data is a substantial increase from previous reviews by Divano et al. [60] and Beckmann et al. [61], which included a combined total of less than 700 cases.

In comparing our findings to those of Divano et al. [60], we observe consistency in the positive outcomes associated with porous metal cones. These authors highlighted the promising results of metal cones in managing bone defects, emphasizing their growing popularity among orthopedic surgeons, which is corroborated by our temporal trend analysis showing an increase in the utilization of metal cones since 2012 [60]. However, while Divano et al. reported generally favorable outcomes, our meta-analysis provides a more granular view by quantifying the pooled incidence of re-revision due to aseptic loosening from a formal meta-analysis. It is worth noting that the recent advancements in metal cone technology might have contributed to these positive outcomes, suggesting an evolution in the management of bone defects.

In contrast, Beckmann et al. [61] focused on the use of structural allografts for severe bone defects, presenting a systematic review that explored their effectiveness and limitations. These authors identified a relatively wide range of outcomes and complications, including graft resorption and nonunion, which aligns with the higher incidence of re-revision observed in our allograft cohort. This similarity underscores the inherent challenges associated with the use of allografts, despite their utility in certain clinical scenarios.

The methodological rigor of our systematic review is underscored by the inclusion of recent studies that reflect current surgical techniques and implant designs. The emergence of second-generation 3D-printed titanium cones, which our review captures, represents a significant innovation in the field since the publication of Divano and Beckmann's reviews [60,61]. Our analysis, therefore, not only encompasses a broader time span but also the evolution of technology, offering an up-to-date assessment of current clinical practices [47].

Subgroup analyses based on follow-up duration and study size provided insights into factors contributing to heterogeneity across studies. While heterogeneity persisted within subgroups, longer follow-up periods did not significantly increase the incidence of re-revision, which could suggest the durability of the metal cones over time. Conversely, larger studies reported higher incidences of re-revision, potentially reflecting the complexity of cases managed in higher volume centers or the increased likelihood of reporting negative outcomes [15,38,46,47].

The clinical implications of our findings suggest that surgeons may prefer porous metal cones due to their consistent outcomes and ease of use. Allografts, sourced from various tissues such as femoral heads, humeral heads, and tibial segments, exhibit considerable variability in how they are processed and prepared [62-64]. This contrasts with metal cones, which are more uniform, particularly with the introduction of second-generation 3D-printed titanium cones [65]. This trend is also evident in the broader literature, where porous metal cones are increasingly recognized for their ability to adapt to a variety of defect sizes and shapes, providing reliable results across different clinical scenarios [66,67].

While our study provides valuable insights, it is not without limitations. The high heterogeneity observed within the metal cones studies indicates that there may be other unmeasured factors contributing to the variability in outcomes. This calls for more robust and standardized reporting in future research, as well as controlled comparative studies to establish clearer evidence of the superiority of one approach over the other. The studies involving porous metal cones tend to be more recent and align with improvements in surgical techniques and technologies for rTKA, which could influence the observed outcomes. Furthermore, the longer follow-up period in allograft studies compared to metal cone studies (83 vs. 53 months) suggests that sustained outcomes must be examined over extended periods to truly understand the long-term success of these interventions. Because aseptic loosening is a time-dependent failure mode, the longer surveillance period in the allograft cohort may have captured a greater cumulative number of late failures, which could partly explain--rather than solely reflect a true difference in implant performance-- the higher re-revision rate observed for allografts. A formal meta-regression to adjust for this and other study-level covariates could not be reliably performed given the limited number of aseptic loosening events relative to the number of candidate covariates and remains a priority for future updated reviews as more studies accumulate. Longitudinal studies with follow-up periods extending beyond ten years would be invaluable in drawing more definitive conclusions. The predominance of retrospective case series and cohort studies within our review points to the inherent challenges of conducting randomized controlled trials (RCTs) in this complex patient population. Nonetheless, the continued evolution of both surgical techniques and implant technology necessitates ongoing research to ensure that findings remain relevant to current practice.

The decision-making process in rTKA for severe bone loss remains multifactorial, involving considerations of patient factors, defect characteristics, surgeon experience, and the availability of cones or allografts in the surgeon's practice [2]. While our review provides a comprehensive analysis of the available literature, the choice between metal cones and allografts should be individualized, with an understanding of the nuances and limitations of each approach. Future research should focus on the long-term clinical outcomes, patient-reported outcomes, and cost-effectiveness of metal cones and allografts to inform best practices. Additionally, the development of national registries could facilitate the collection of large-scale, high-quality data to further elucidate the comparative effectiveness of these interventions.

## Conclusions

This systematic review and meta-analysis demonstrate that porous metal cones result in lower re-revision rates due to aseptic loosening compared to structural allografts in revision total knee arthroplasty (rTKA). These findings suggest that porous metal cones should be considered preferentially in complex rTKA cases, supported by their consistent performance. Clinicians are advised to integrate these insights into their decision-making process, particularly for long-term stability and reliability. Future studies should aim to extend these findings through long-term longitudinal research and randomized controlled trials to solidify treatment protocols.

## Appendices

Appendix 1 

**Table 2:** Search strategy by database.

Database	Search concept	Search terms/Boolean combination
MEDLINE	Revision TKA	"Revision TKA" OR "Revision Total Knee Arthroplasty" OR "Revision Total Knee Replacement"
MEDLINE	Structural Allografts	"Structural Allografts" OR "Bone Grafts" OR "Allograft Reconstruction"
MEDLINE	Porous Metal Cones	"Porous Metal Cones" OR "Porous Tantalum Cones" OR "Trabecular Metal Cones" OR "Metal Augments"
MEDLINE	Combined search	(Revision TKA OR Revision Total Knee Arthroplasty OR Revision Total Knee Replacement) AND (Structural Allografts OR Bone Grafts OR Allograft Reconstruction) AND (Porous Metal Cones OR Porous Tantalum Cones OR Trabecular Metal Cones OR Metal Augments)
EMBASE	Revision TKA	'revision TKA' OR 'revision total knee arthroplasty' OR 'revision total knee replacement'
EMBASE	Structural Allografts	'structural allograft' OR 'bone graft' OR 'allograft reconstruction'
EMBASE	Porous Metal Cones	'porous metal cone' OR 'porous tantalum cone' OR 'trabecular metal cone' OR 'metal augment'
EMBASE	Combined search	('revision TKA' OR 'revision total knee arthroplasty' OR 'revision total knee replacement') AND ('structural allograft' OR 'bone graft' OR 'allograft reconstruction') AND ('porous metal cone' OR 'porous tantalum cone' OR 'trabecular metal cone' OR 'metal augment')
CINAHL	Revision TKA	"Revision TKA" OR "Revision Total Knee Arthroplasty" OR "Revision Total Knee Replacement"
CINAHL	Structural Allografts	"Structural Allografts" OR "Bone Grafts" OR "Allograft Reconstruction"
CINAHL	Porous Metal Cones	"Porous Metal Cones" OR "Porous Tantalum Cones" OR "Trabecular Metal Cones" OR "Metal Augments"
CINAHL	Combined search	(Revision TKA OR Revision Total Knee Arthroplasty OR Revision Total Knee Replacement) AND (Structural Allografts OR Bone Grafts OR Allograft Reconstruction) AND (Porous Metal Cones OR Porous Tantalum Cones OR Trabecular Metal Cones OR Metal Augments)

Note: Each search was limited to studies published up to June 30, 2023. No language restrictions were applied.		

Appendix 2 

**Table 3:** Risk of bias and methodological quality assessment domains. We utilized the risk of bias tool developed by Luijken et al. [8], which incorporates elements from the Cochrane Risk of Bias 2 (RoB-2) tool, the Risk Of Bias In Non-randomized Studies of Interventions (ROBINS-I) tool, and the Methodological Index for Non-Randomized Studies (MINORS) criteria. This tool is specifically tailored for assessing comparative studies of operative interventions in orthopedic trauma surgery. The tool consists of nine key items split into two subsets: applicability and methodological quality, each assessed using tailored signaling questions.

Category	Domain	Description/Signaling question focus
Applicability	Population	Assess if the study population matches the clinical setting and patient demographics relevant to the research question. Key elements include anatomical location of the condition, type of injury, and age group.
Applicability	Intervention	Evaluate whether the intervention is described in sufficient detail, including surgical technique, pre- and post-operative care, and surgeon experience.
Applicability	Comparator	Determine if the comparator is relevant and adequately described. This may include alternative surgical techniques or non-surgical interventions.
Applicability	Outcome	Assess the appropriateness and clarity of the outcome measures, including how and when outcomes are measured.
Methodological Quality	Confounding	Evaluate the management of potential confounding variables. In randomized studies, this involves assessing randomization and allocation concealment. In observational studies, it includes statistical adjustment for confounders.
Methodological Quality	Missing Data and Selection Bias	Assess how the study handles missing data and the potential for selection bias. This includes describing the reasons for missing data, the methods used to handle it, and assessing the representativeness of the study sample.
Methodological Quality	Intervention Status	Verify the clarity of intervention status classification, ensuring accurate differentiation between groups receiving different interventions.
Methodological Quality	Outcome Assessment	Assess the validity and reliability of outcome assessment methods, including blinding of outcome assessors and use of validated measurement tools.
Methodological Quality	Pre-specification of Analysis	Evaluate the extent to which the statistical analysis plan was pre-specified, including handling of multiple testing and adjustments.
